# Molecular and Cellular Mechanisms of the Therapeutic Effect of Mesenchymal Stem Cells and Extracellular Vesicles in Corneal Regeneration

**DOI:** 10.3390/ijms252011121

**Published:** 2024-10-16

**Authors:** Nina Kobal, Miha Marzidovšek, Petra Schollmayer, Elvira Maličev, Marko Hawlina, Zala Lužnik Marzidovšek

**Affiliations:** 1 Eye Hospital, University Medical Centre Ljubljana, 1000 Ljubljana, Slovenia; nina.kobal@kclj.si (N.K.);; 2 Blood Transfusion Centre of Slovenia, 1000 Ljubljana, Slovenia; 3 Biotechnical Faculty, University of Ljubljana, 1000 Ljubljana, Slovenia; 4 Medical Faculty, Department of Ophthalmology, University of Ljubljana, 1000 Ljubljana, Slovenia

**Keywords:** mesenchymal stem cells, extracellular vesicles, corneal regeneration, cell-based therapy, corneal epithelium, corneal stroma, dry eye disease, limbal stem cell deficiency, keratoconus, immune and inflammatory modulation

## Abstract

The cornea is a vital component of the visual system, and its integrity is crucial for optimal vision. Damage to the cornea resulting from trauma, infection, or disease can lead to blindness. Corneal regeneration using mesenchymal stem cells (MSCs) and MSC-derived extracellular vesicles (MSC-EVs) offers a promising alternative to corneal transplantation. MSCs are multipotent stromal cells that can differentiate into various cell types, including corneal cells. They can also secrete a variety of anti-inflammatory cytokines and several growth factors, promoting wound healing and tissue reconstruction. This review summarizes the current understanding of the molecular and cellular mechanisms by which MSCs and MSC-EVs contribute to corneal regeneration. It discusses the potential of MSCs and MSC-EV for treating various corneal diseases, including corneal epithelial defects, dry eye disease, and keratoconus. The review also highlights finalized human clinical trials investigating the safety and efficacy of MSC-based therapy in corneal regeneration. The therapeutic potential of MSCs and MSC-EVs for corneal regeneration is promising; however, further research is needed to optimize their clinical application.

## 1. Introduction

**Figure 1:**
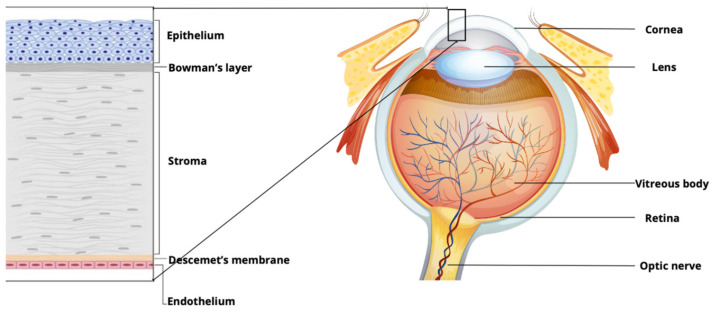
Schematic cross-section of a human eye with an expanded view of the cornea.

The cornea, an avascular and immune-privileged tissue, is one of the main components of the visual system [1,2]. It is the outermost transparent layer of the eye, and it plays an important role in transmitting and refracting light to the retina. Corneal tissue is made up of five layers (Figure 1): the epithelium, followed by Bowman’s membrane, avascular corneal stroma, Descemet’s membrane (DM) and the innermost corneal endothelium [1].

The corneal epithelium is the outermost part. It is composed of five to seven layers of stratified non-keratinized squamous cells [3]. A healthy corneal epithelium is of vital role for the maintenance of corneal integrity and transparency [4]. The stroma is the thickest corneal layer, consisting of the extracellular matrix (ECM) and collagen fibrils that are organized into flattened lamellae that run perpendicular to each other. Stromal keratocytes are typically located between the lamellae. The cornea remains transparent and biomechanically strong due to the precise organization of collagen fibrils [1]. The corneal endothelium is a single neural crest-derived cell layer. It forms a barrier between the stroma and the anterior chamber [1]. Endothelial cells transport fluid from the stromal layer to the anterior chamber, keeping the cornea in relatively dehydrated state and clear [1]. Human corneal endothelial cells (CEnCs) have limited ability to undergo cell division in vivo, and a gradual decline in CEnCs density is observed during adulthood due to age-related cell death [1,5]. In case of substantial CEnC loss or dysfunction, pathological corneal hydration (called ‘edema’) occurs, leading to visual impairment [1,6].

Severe corneal damage can occur as a consequence of several clinical conditions, such as trauma or chemical injuries, infections, systemic diseases, degenerations and corneal dystrophies. All these factors, along with inefficient tissue-repair processes, trigger corneal scarring and neovascularization that can lead to a complete loss of vision, compromising the patient’s quality of life and putting an immediate burden on the healthcare systems [1,7]. Corneal diseases thus represent the fifth leading cause of blindness worldwide with approximately 4.5 million individuals being visually impaired due to loss of corneal clarity [7].

**Figure 2:**
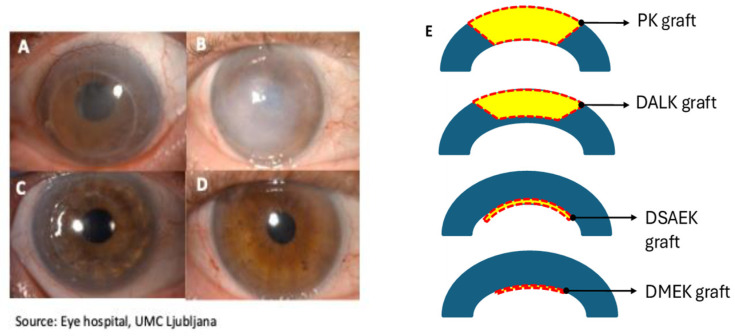
Various corneal pathologies can lead to corneal edema and opacification (**A**,**B**), which is conventionally treated by surgical removal and donor corneal transplantation (penetrating (**C**) and lamellar keratoplasties (**D**)). (**A**) Bullous keratopathy and corneal scar years after penetrating injury and (**C**) 1 month after PK. (**B**) Bullous keratopathy years after cataract surgery and (**D**) 1 month after DMEK. (**E**) A schematic representation of different types of corneal transplantation techniques. The blue section represents the recipient cornea and the yellow section in the red square–dot line represents the transplanted donor corneal graft tissue. In PK, all corneal layers are transplanted, whereas in DALK, only the anterior corneal layers are transplanted. Posterior lamellar techniques involve selective removal of the patient’s Descemet membrane (DM) and endothelium, which is followed by either the transplantation of the donor corneal endothelium, the DM and a thin stromal layer in DSAEK or by the transplantation of only the donor DM and the endothelium in DMEK. Abbreviations: PK—penetrating keratoplasty; DALK—deep anterior lamellar keratoplasty; DSAEK—Descemet’s stripping automated endothelial keratoplasty; DMEK—Descemet’s membrane endothelial keratoplasty.

The diseased or damaged corneal layers can be replaced by healthy donated corneal tissue with corneal transplantation, which remains the most common form of solid tissue transplantation [1,8]. However, despite significant advances in surgical techniques (Figure 2) and tissue storage methods, there are still major issues related to the availability and quality of donated corneal tissues and postoperative corneal transplant survival. Although the number of donor corneas increases steadily, the supply of transplantable tissue is consistently insufficient as the demand surpasses the availability. Currently, approximately 13 million people worldwide need corneal transplantation [1,9]. A shortage of donor tissues, allograft survival, the prolonged use of immunosuppressive therapy after transplantation, the need for specialized corneal centers, and religious and ethical dilemmas are all the reasons why we need alternative treatment options with regenerative cell-based medicine being one of them. The cornea is well suited for regenerative tissue therapy because it is immune privileged and avascular, which makes it less likely to reject transplanted cells compared to other organs [1,10]. Moreover, due to its relative ease of accessibility and the non-invasive diagnostic methods to follow up and visualize eye structures after therapy (e.g., slit-lamp examination, optical coherence tomography, in vivo confocal microscopy), the human eye is a prime target for stem cell-based therapy development.

Thus, extensive research effort is being put into the development of new regenerative therapeutic options such as advanced therapy medicinal products (ATMPs). ATMPs are medical products based on genes, tissues or cells [11], including various stem cells (such as mesenchymal stem cells) and extracellular vesicle (EV) therapies [11,12]. These new stem cell-based therapies offer the possibility of permanently restoring or replacing previously irreparable tissues or organs and have several well-described paracrine functions that can prevent or halt disease progression.

In this review, we summarize the current understanding of the molecular and cellular mechanisms by which mesenchymal stem cells (MSCs) and MSC-derived EVs (MSC-EVs) contribute to corneal regeneration. We discuss the potential of MSCs and MSC-EVs for treating various corneal diseases, including corneal epithelial defects, dry eye disease (DED), and keratoconus with the support of knowledge from finalized clinical trials investigating the safety and efficacy of MSCs and MSC-EVs in corneal regeneration.

## 2. Mesenchymal Stem Cells

Stem cells (SCs) are undifferentiated cells that have not yet committed to a specific lineage. They possess the ability to divide and differentiate into various mature and functional cell types [13]. Among these, one of the most important adult SC sources is multipotent MSCs. MSCs exist in various tissues throughout the developmental process. They play a vital role in tissue repair and regeneration and can modulate immune responses via paracrine function [14,15]. To date, they are the most commonly used SCs in clinical trials (https://clinicaltrials.gov/ (accessed on 15 June 2024)) and can be isolated from various adult as well as fetal tissues (such as placenta, Wharton’s jelly and umbilical cord blood) [15].

MSCs can be classified into several categories based on various criteria. One common classification is based on the MSCs source, which includes bone marrow-derived MSCs (MSCs(M)), adipose tissue-derived MSCs (MSCs(AT)), and umbilical cord blood-derived MSCs (MSCs(CB)); other sources of MSCs are also dental pulp, liver, spinal cord, placenta, skeletal muscle, synovium and periosteum [16]. In addition, corneal stromal SCs (CSSCs) are a distinct MSC population situated in the anterior stroma near the limbal niche; thus, they are also called limbus-derived MSCs by some authors [17]. They act as a reservoir of progenitor cells capable of differentiating into functional keratocytes, which produce and organize the ECM [18]. According to their in vitro characteristics, they can be characterized as MSCs [19]. Both CSSCs and MSCs have the potential to differentiate into various cell types, but there are notable differences between them as they each display specific lineage commitments [18]. CSSCs primarily differentiate into corneal keratocytes, which play a crucial role in maintaining the corneal stroma [18]. On the other hand, MSCs are a more broadly distributed population found in various tissues in the human body [18]. Fetal MSCs have more primitive characteristics, more active telomeres and a better ability to grow compared to adult MSCs. However, to obtain enough of these cells, they need to be grown and multiplied outside the body after their isolation, which can reduce their effectiveness [1,20].

The Mesenchymal and Tissue Stem Cell Committee of the International Society for Cellular Therapy (ISCT) has established specific criteria for defining human MSCs to ensure consistency and reliability in research and clinical applications. According to the ISCT, MSCs must meet three primary criteria. Firstly, MSCs must adhere to plastic surfaces under standard culture conditions, indicating their ability to proliferate in vitro. Secondly, these cells must express CD105, CD73, and CD90 while lacking the expression of CD45, CD34, CD14, CD11b, CD79alpha, CD19, and human leukocyte antigen (HLA)-DR surface molecules. Lastly, MSCs should be able to differentiate in vitro into osteoblasts, adipocytes, and chondrocytes [21]. In addition, MSCs can be classified as autologous (obtained from the same individual receiving the treatment) or allogeneic (obtained from a different individual) [21].

Endogenous MSCs(M) can migrate to injury sites, where they proliferate, differentiate, and secrete various anti-inflammatory and growth factors that can promote wound healing and thus reconstruct the damaged tissue [22]. While there are no MSCs(M) in the healthy cornea, specific chemical mediators can stimulate endogenous MSCs(M) mobilization following corneal injury [23]. Activated MSCs(M) enter the peripheral bloodstream and travel to the site of injury in the cornea, where they can promote corneal regeneration [23,24]. Moreover, locally present CSSCs can also quickly respond to corneal injury by differentiating into functional keratocytes and are essential for maintaining corneal avascularity and immune privilege [18]. However, if there are not enough CSSCs available, MSCs(M) can be used to some extent in formulating therapeutic cell sources [18].

In addition, MSCs have various paracrine effects that result in therapeutic impact [1]. They secrete many immunomodulatory cytokines related to tissue repair, including vascular endothelial growth factor (VEGF), fibroblast growth factor 2 (FGF-2), insulin-like growth factor 1 (IGF-1), and hepatocyte growth factor (HGF) [25]. Among those, HGF is especially important, as it was shown that it can inhibit the generation of opacity-inducing myofibroblasts [24]. Mittal et al. reported that HGF alone was able to restore corneal transparency in an in vivo model of eye injury [24].

MSCs express low levels of major histocompatibility complex (MHC) I and MHC II molecules without their co-stimulatory molecules [26]. These features allow MSCs to be immune evasive [27]. MSCs are covered by glycocalyx with a high content of anti-inflammatory factors (TSG-6, pentraxin-3) [28]. These factors are involved in regulating the host’s inflammatory response after transplantation [28]. Thus, in case of allogeneic transplantation, MSCs were thought to avoid immune rejection [29]. However, recent studies have revealed the generation of antibodies against allogenic MSCs and the occurrence of immune rejection, indicating that MSCs do not possess complete immune privilege [27].

The route of MSCs administration depends on the specific condition being treated, the target tissue, and the desired therapeutic effect. Each method has its benefits and drawbacks, and careful consideration is necessary to optimize treatment outcomes. So far, researchers have explored two primary methods of administering MSCs for corneal disease treatment: intravenous injection and local application, which includes tissue transplants, anterior chamber injection, periorbital injection, and topical eye drop application [1]. Intravenous administration is suitable for treating systemic diseases as it allows the widespread distribution of MSCs throughout the body [30,31]. However, MSCs may become trapped in the lungs or other organs, reducing effectiveness at the target site. The administration of MSCs directly to the damaged area of the cornea (e.g., using subconjunctival injections or transplantation with the amniotic membrane) could result in an increased MSC concentration at the site of injury and consequently greater efficacy and better outcomes [32,33].

## 3. Corneal Regeneration by MSCs Differentiation into Corneal Cells

**Figure 3:**
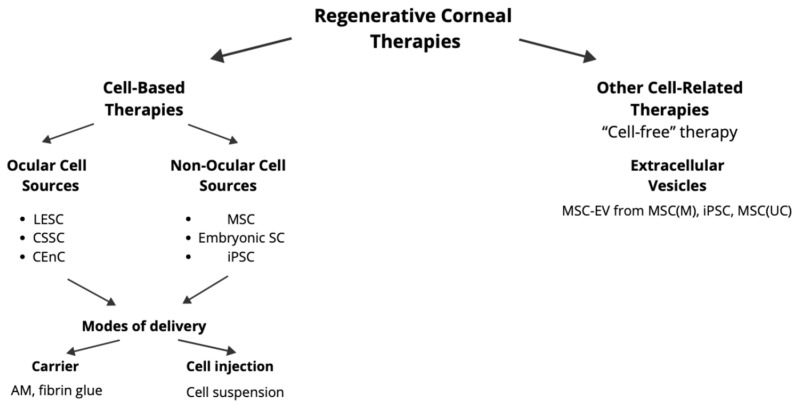
Schematic representation of different regenerative corneal therapies. Abbreviations: LESC—limbal epithelial stem cell; CSSC—corneal stromal stem cell; CEnC—corneal endothelial cell; MSC—mesenchymal stem cell; SC—stem cell; iPSC—induced pluripotent stem cell; AM—amniotic membrane; MSC-EV—extracellular vesicle derived from mesenchymal stem cell; MSC(M)—bone marrow-derived mesenchymal stem cell; MSC(UC)—umbilical cord-derived mesenchymal stem cell.

The field of regenerative medicine using MSC-based therapies holds immense promise for the treatment of corneal diseases. This new paradigm, which focuses on tissue regeneration instead of tissue replacement, may revolutionize the current clinical practice. As presented in Figure 3, regenerative corneal therapies can be divided into cell-based therapies and cell-related therapies, also called “cell-free” therapies, which include therapies with EVs and are derived from cells. MSCs are an extra-ocular multipotent SC source and may promote corneal tissue regeneration via the paracrine function by secreting anti-inflammatory, anti-fibrotic, and anti-apoptotic growth factors and cytokines or by direct differentiation into corneal cells, as presented in the below paragraphs.

### 3.1. Corneal Epithelial Regeneration

The corneal epithelium is a non-keratinized squamous epithelium, which is renewed by a small population of adult corneal epithelial SCs primarily found at the peripheral corneal area, the limbus (thus also named limbal epithelial SCs). Corneal epithelial failure due to severe limbal stem cell deficiency (LSCD) is an end-stage pathology resulting from multiple diseases that destroy the corneal epithelium SC niche. MSCs can differentiate into cell lineages derived from the neuroectoderm and epithelial cells [34].

Various studies showed that MSCs can differentiate into corneal epithelial cells both in vitro and in vivo, displaying morphology similar to epithelial cells and expressing cytokeratin 3 and 12, which are specific corneal epithelial markers [34,35,36]. In vivo experiments showed reduced corneal opacity, neovascularization and a decline in inflammation markers after MSCs transplantation [37,38].

### 3.2. Corneal Stromal Regeneration

The corneal stroma represents around 90% of corneal thickness and is composed of parallel collagen fibers and interspersed scarce keratocytes, which are responsible for the production and organization of the stromal extracellular matrix. The precise spacing of collagen fibers is essential for stromal transparency. Stromal keratocytes remain quiescent throughout life. They are derived from the embryonic periocular mesenchyme that originates from the neural crest [18].

After injury or severe corneal infections, the accompanying inflammatory response can cause stromal keratocytes to undergo apoptosis [39]. This results in a reduced production of stromal proteoglycans, degradation of collagen fibrils, and increased glycation of collagen fibrils. Inflammation may, on the other hand, activate and transform some surviving keratocytes into repair-type stromal fibroblasts and highly contractile myofibroblasts initiating a wound-healing response, which ultimately results in the formation of corneal scars [18]. Corneal scars disrupt the transmission of light and worsen vision [1].

Studies have shown that MSCs obtained from bone marrow and the umbilical cord can transform into keratocyte-like cells and potentially restore corneal stromal transparency [33,40,41]. Researchers observed a restoration of physiologic stromal anatomy (e.g., improvement in collagen structure, restoration of corneal thickness and transparency after the transplantation of MSCs(CB)) [40]. Furthermore, MSCs downregulated inflammatory cytokines, resulting in a lower risk of rejection to transplanted cells [40]. When MSCs obtained from bone marrow, adipose tissue, dental pulp and limbal stroma were cultured under conditions that promoted keratocyte differentiation, genes associated with corneal stromal keratocytes were upregulated at the RNA and protein levels, suggesting MSCs started showing similar characteristics to keratocytes [33,42,43]. The transplantation of MSCs derived from dental pulp (MSCs(DP)) into rat eyes induced the production of a stromal ECM consisting of collagen type I and keratocan, suggesting that MSCs(DP) can differentiate into stromal keratocytes and are therefore easy to obtain and a safe therapeutic option for maintaining corneal transparency [44].

Corneal stromal stem cells (CSSCs) derived from the limbal stroma share many characteristics with MSCs, however, with more specific differentiation potentials [45,46]. They serve as a reservoir of progenitor cells that can differentiate into functional keratocytes, which are responsible for producing and organizing the ECM [18]. CSSCs respond rapidly to corneal injury and also play a vital role in preserving corneal avascularity and immune privilege [18]. Recent studies observed that CSSCs can differentiate into corneal stromal keratocytes when grown in a serum free-environment that has been enriched with basic fibroblast growth factor (FGF) and transforming growth factor β3 (TGFβ3). This suggests that CSSCs have the potential for stromal regeneration as they are able to deposit an ECM similar to that of the native stroma [47,48].

### 3.3. Corneal Endothelial Regeneration

One of the primary functions of the corneal endothelium is maintaining corneal stromal dehydration and clarity. CEnCs have a limited ability to undergo cell division in vivo [1,5]. Any dysfunction of the endothelium results in corneal edema (called clinically bullous keratopathy), which leads to visual impairment [1].

To date, the best treatment option is corneal transplantation, which enables a fast visual rehabilitation in patients with corneal endothelial disease [49]. However, in many parts of the world, a shortage of donor corneas limits access to treatment, prompting a search for alternatives.

MSCs may represent a promising approach for replacing the corneal endothelium [1,50]. In one study, they were investigating the effect of a conditioned medium (CM) obtained from MSCs(M) on human CEnCs in cultures. The results of the study suggest that when treated with a CM obtained from MSCs(M), CEnCs retain the required proliferative potential with the capacity to be fully differentiated [5]. In another animal study on rabbits, a damaged endothelium was restored by the transplantation of MSCs(M). Researchers observed that the transplanted MSCs were differentiated into a single layer of irregular-shaped cells with similarity to polygonal-shaped cells [51]. Yamashita et al. induced the differentiation of MSCs(CB) with medium containing glycogen synthase kinase (GSK) 3-β inhibitor. MSCs(CB) began to form polygonal CEnC-like cells that functioned as a tissue-engineered corneal endothelium (UTECE). They confirmed the expressions of major functional and developmental markers of CEnCs. When the UTECE was transplanted into a rabbit model of bullous keratopathy, it successfully maintained corneal thickness and transparency [52]. Recently, it was found that human MSCs from Wharton jelly (MSCs(WJ)) can differentiate into cells similar to corneal endothelial cells [53]. Researchers observed an increase in the expression of genes specific for endothelial cells (e.g., COL-8, ZO-1, Na/K-ATP-ase) after they implanted MSCs(WJ) on a denuded Descemet membrane to create an endothelium-like layer [54].

## 4. Corneal Regeneration by MSC Paracrine Function

The therapeutic effect of MSCs can be attributed to the secretion of soluble factors that regulate tissue wound repair, inflammation, angiogenesis and immune responses. MSCs are known to regulate various immune cells and have immune regulatory and anti-inflammatory effects [1]. Moreover, if MSCs are exposed to various pro-inflammatory factors like TNF-α and IL-1α, their immunosuppressive properties are markedly enhanced, resulting in their differentiation into an immunosuppressive phenotype [55].

### 4.1. Suppression of Corneal Inflammation

MSCs exhibit potent anti-inflammatory effects, which are particularly beneficial in the context of corneal diseases and injuries. When MSCs enter corneal tissue, they decrease the infiltration of inflammatory cells and macrophages that express CD68 [1]. They can modulate immune responses by secreting a variety of anti-inflammatory cytokines, such as interleukin-10 (IL-10), TGF-β and prostaglandin E2 (PGE2), which help to inhibit the activation of pro-inflammatory cells and pathways [31,38]. Studies have shown that MSCs can reduce the inflammatory response in corneal tissue by downregulating the secretion of pro-inflammatory cytokines, such as interleukin-1 (IL-1), interleukin-2 (IL-2) and tumor necrosis factor-alpha (TNF-α). This immunomodulatory capacity not only prevents excessive inflammation during corneal injuries but also promotes tissue healing and regeneration. Furthermore, the application of MSCs in corneal transplantation has demonstrated a reduction in graft rejection rates, which is attributed to their ability to maintain an anti-inflammatory environment [1,31,38,56]. However, some recent studies suggest that MSCs do not have complete immune privilege, as they revealed the generation of antibodies against allogeneic MSCs [27].

### 4.2. Inhibition of Corneal Neovascularization

The cornea is characterized as an avascular tissue, meaning it lacks a direct blood supply. This absence of blood vessels contributes to its transparency, which is crucial for optimal visual function, and immune privilege. MSCs have shown significant potential in inhibiting corneal neovascularization. The anti-angiogenic properties of MSCs are attributed to their ability to secrete a variety of cytokines and growth factors that inhibit abnormal blood vessels formation [1,34]. Studies have demonstrated that MSCs can downregulate pro-angiogenic factors, such as VEGF, while enhancing the expression of anti-angiogenic factors (pentraxin-3 and thrombospondin-1) [1,57,58]. Additionally, MSCs are capable of modulating immune responses and creating a microenvironment that favors tissue repair without promoting neovascularization [1,38]. This makes them a promising therapeutic option in conditions such as corneal neovascularization and graft rejection. Furthermore, MSCs can express pro- and anti-angiogenic factors, depending on the tissue microenvironment. They can promote angiogenesis in certain tissues while inhibiting it in others (e.g., in cornea) [59].

### 4.3. Corneal Immune-Privilege and Influence of MSCs on Transplant Immunity

The cornea is considered an immune-privileged tissue, allowing it to tolerate foreign antigens without provoking an inflammatory response [1,2]. Immune privilege is the result of an actively maintained immunosuppressive response to ocular antigens, which was later referred to as an anterior chamber-associated immune deviation (ACAID) [60]. It is a form of immune tolerance to alloantigens placed in the anterior chamber of the eye that results in the downregulation of an antigen-specific delayed hypersensitivity response while promoting humoral immunity and the production of non-complement fixing antibodies [61]. Aside from ACAID, several other mechanisms contribute to the maintenance of corneal immune privilege with the cornea expressing various membrane-bound immunomodulatory molecules that protect it from inflammation and promote immune inactivity [60].

Therefore, immune rejections to corneal allografts occur less often compared to other solid organs even without systemic immunosuppression. However, in case of advanced corneal diseases, this immune-privilege is often lost, and allograft rejection in high-risk recipients still represents a major issue [62]. Patients with a previous history of graft rejection or grafts performed in inflamed and vascularized host beds are considered at high risk of rejection, while non-vascularized and uninflamed host beds are regarded as low risk [60]. Interferon-γ (IFN-γ)-producing CD4+ Th1 cells are considered to be the predominant effector cells in corneal graft rejection, although the exact mechanisms are not yet fully understood [60].

MSCs have immunomodulatory properties [26]. In vitro studies showed that MSCs affect the innate immune system by suppressing the maturation and activation of dendritic cells as well as the cytotoxicity of natural killer cells [63]. They also suppress the adaptive immune system by inhibiting the proliferation and secretion of cytokines by T cells and maturation of B cells [63]. In addition, MSCs’ immunosuppressive function includes various soluble factors such as TGFβ, IL-10, matrix metalloproteinases (MMPs), PGE2, indoleamine-2, 3-dioxygenase (IDO), HLA-G5 and nitric oxide [64]. MSCs can suppress the production of IFN-γ by Th1 cells while increasing the production of IL-4 and IL-10 by Th2 cells, which results in promoting a shift toward a Th2-type of immune responses [65].

Thus, MSCs could prevent allograft immune rejection as they can inhibit the release of pro-inflammatory cytokines by T-cells [28,66]. By regulating the generation of regulatory T-cells, MSCs could support immune tolerance and promote graft survival [28,66]. MSCs can also induce cell cycle arrest, which results in the inability of activated T cells to divide [67]. Some animal studies have demonstrated that MSCs lead to the suppression of inflammation and decreased activation of antigen-presenting cells in the cornea, resulting in an increased survival rate of allografts and a reduced risk of immune rejection [56,68,69].

## 5. MSC-Derived Extracellular Vesicles

**Figure 4:**
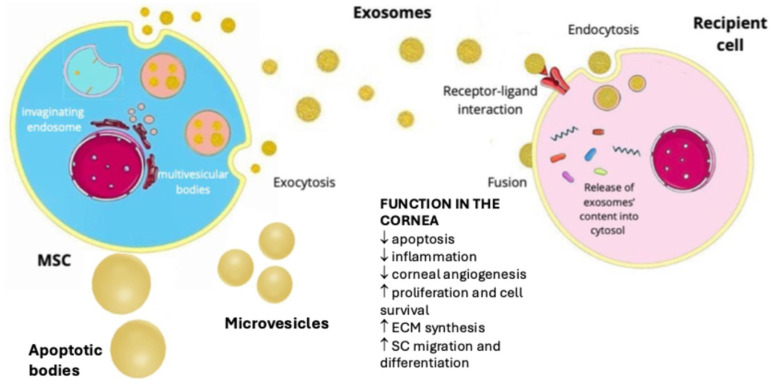
Schematic representation of mesenchymal stem cell-derived extracellular vesicles (such as exosomes, microvesicles and apoptotic bodies), their biogenesis and transfer from cell of origin to recipient cell. They are capable of transferring bioactive molecules to recipient cells through three different mechanisms: endocytosis, specific receptor–ligand interactions and direct fusion. Extracellular vesicles have various effects on corneal cells. Abbreviations: MSC—mesenchymal stem cell; ECM—extracellular matrix; SC—stem cell.

EVs are cell-derived lipid membrane vesicles of varying sizes that can be secreted by many cell types [70,71,72]. Depending on the size and their biogenesis, they can be classified as (1) exosomes (30–150 nm in diameter), which are released into the extracellular space by the intracellular budding of endosomes; (2) microvesicles (100–1000 nm in diameter), which are formed by the budding of the cell membrane; and (3) apoptotic bodies (1000–5000 nm in diameter) [70]. After being secreted into the extracellular space, EVs can be internalized by recipient cells in the local microenvironment or transported to distant regions via the circulatory system [73]. The uptake of EVs by target cells can occur through three mechanisms: (a) endocytosis, which is a cellular process through which substances are brought into the cell with the cell membrane engulfing extracellular material, forming a vesicle that is then internalized; (b) interactions between ligands and receptors, which refer to the binding of the ligand to a specific receptor on a cell’s surface, which triggers a cellular response, and (c) direct fusion with the cell membrane (Figure 4) [73]. Once merged, their content is released into the cytoplasmic space of the target cell.

EVs carry important bioactive molecules, such as cytokines, growth factors, signaling lipids, messenger ribonucleic acids (mRNAs) and regulatory microribonucleic acids (miRNAs), that are involved in intracellular communication and several signaling cascades [74]. The composition of their cargo is highly variable and depends on the cell type of origin and current conditions in the environment in which they were formed [1]. EVs can influence a wide variety of biological functions, such as cell proliferation, regeneration, migration, apoptosis, and immunoregulation [74]. Their final function depends on the types of nucleic acids, proteins, and lipids they contain [1]. By transferring mRNA or miRNA, EVs can influence new protein synthesis and modulate gene expression [74].

It has been shown that MSCs produce a greater quantity of EVs compared to other cells [75]. MSC-EVs display commonly occurring surface proteins and contain diverse types of nucleic acids, including miRNA and mRNA. In particular, miRNAs are crucial components as they are involved in several important biological mechanism, such as cell differentiation, angiogenesis, apoptosis and inflammatory pathways which can all significantly influence the wound-healing process [1]. EVs are supposed to have similar paracrine therapeutic effects as the original SCs; thus, they could be safer to use clinically due to their cell-free nature and can also be administered in higher concentrations, which results in a higher bioavailability [76]. They have a lower risk of immunological rejection, uncontrolled cell proliferation and tumor formation compared to cell-based therapies [1]. They can freely pass through various biological barriers without blocking microvascular circulation, which is often observed in systemic MSC treatment, and are therefore safer and may have better pharmacological profiles [1,77]. However, although the therapeutic potential of exosomes (and other subtypes of EVs) is promising, the reproducibility, vesicle integrity and maintenance of their biological activity to ensure the final product homogeneity remains challenging [71]. Thus, to update the experimental requirements for the definition of EVs and their functions, new Minimal Information for Studies of Extracellular Vesicles (MISEV) 2023 guidelines have been published [78].

Numerous studies in animal models have been conducted examining the influence of EVs (mostly exosomes) on corneal epithelial healing [79,80,81,82,83,84,85,86,87]. In those studies, researchers described various effects of exosomes on promoting the proliferation of corneal epithelial cells, reduced fibrosis and inflammation. This includes lowering levels of pro-inflammatory cytokines like TNF-α, IL-1β, and IL-6 while increasing anti-inflammatory cytokines such as IL-10 and TGF-β. They also observed less scar formation and corneal haze. Cell proliferation and progression through the cell cycle was improved. Additionally, there was a reduction in the expression of genes related to cell death, such as Bax and caspase [79,80,81,82,83,84,85,86,87]. In another in vitro study where rabbit corneal keratocytes were cultured with MSCs(AT) exosomes, there was an increase in cell proliferation, reduced cell death, downregulation of MMPs and upregulation in the synthesis of ECM-related proteins (collagen and fibronectin) [88]. Furthermore, Shen et al. studied the impact of exosomal miRNAs obtained from MSCs(AT) on the differentiation process of rabbit corneal keratocytes. They observed a suppression of corneal keratocytes transformation into myofibroblasts by the inhibition of homeodomain-interacting protein kinase 2 (HIPK2) expression [89].

An in vitro study on CEnCs showed that exosomes were capable of regenerating damaged CEnCs by decreasing the number of apoptotic cells [90]. Thus, we suppose that MSC-derived exosomes (MSC-Exos) may also be important for preserving donor corneas, as they help maintain CEnC health and the corneal structure through their content of growth factors, cytokines and other beneficial molecules that support cell communications and could protect against damage during storage [1,74]. Furthermore, an in vivo study on rats showed that injecting exosomes under the conjunctiva helped extend the survival of the corneal allograft by preventing the infiltration of certain immune cells (CD4+ and CD25+ T-cells) and lowering levels of IFN-γ and chemokine ligand 11 (CXCL11) [91]. No adverse effects were reported.

## 6. Current Clinical Trials Using MSCs and MSC–Extracellular Vesicles for Corneal Regeneration

Based on encouraging results obtained from several preclinical studies on animal models, the first human clinical studies emerged as novel approaches for corneal regeneration. Table 1 summarizes the reported clinical trials and case reports using various MSCs or MSCs-derived EVs and presents their therapeutic effect in treating severe corneal diseases, which is presented in more detail below.

### 6.1. Limbal Stem Cell Deficiency Disease

Severe ocular surface damage may lead to complete vision loss due to ocular surface failure as a result from insufficient corneal epithelial renewal and stromal scaring, which is clinically referred to as LSCD disease [103,104]. LSCD develops due to the loss or dysfunction of limbal epithelial SC and can result from various causes including trauma, chemical burns, autoimmune diseases, infections, and genetic disorders [104]. LSCD results in recurrent corneal epithelial ulceration, neovascularization, and opacification because of the inability of the limbal niche to renew the corneal epithelium [92]. LSCD can be partial or total and can affect one or both eyes. The management of patients with total LSCD requires SC transplantation for corneal epithelial restoration [104].

In cases where only one eye is affected, an autologous limbal graft can be obtained from the healthy fellow eye, although this carries a hypothetical risk of inducing LSCD in the donor eye [104]. For bilateral cases, limbal tissue from a donor is necessary. If no living related donor is available or willing to donate, keratolimbal allograft (KLAL) transplantation can be an option. It uses cadaveric allogeneic limbal tissue as the source of corneal SCs and allows a larger supply of SCs [104]. Nonetheless, the success of KLAL is limited by immune rejections of the allograft tissue, which can occur in more than half of the cases despite aggressive systemic immunosuppressive therapy [34].

As conventional surgical management of patients with total LSCD faces several challenges, another approach using ex vivo cultured SC transplantation has been developed since 1997. Currently, SC-based therapies based on cultivated limbal epithelial cells are a viable option in several corneal centers worldwide and can be prepared from autologous and allogeneic limbal sources, the latter being used when there is no healthy contralateral donor eye [104]. In this method, SCs are typically obtained and cultured from a small limbal biopsy measuring 1 to 2 mm^2^ and taken from the peripheral corneal region (e.g., cultivated limbal epithelial transplantation (CLET)). Limbal epithelial cells can be cultivated and transferred on various scaffolds, the most common being amniotic membrane or fibrin gel.

Another promising and potentially more sustainable SC source in ocular surface reconstruction might be MSCs. MSCs could have potential advantages over limbal epithelial SCs as they can be easily obtained from many tissue types without any dependence on deceased donors [105]. Additionally, they can be cultured in vitro to sufficient clinical scales in a short period of time, overcoming limbal epithelial cell limitations, which are difficult to obtain, isolate, and culture and have limited availability [106]. Furthermore, allogeneic MSCs can be in some cases transplanted without the need for systemic immunosuppression to avoid immune rejection [107].

Although MSCs have been widely studied in various animal disease models [30,36,108,109,110], to date, only a limited number of research teams have presented clinical findings regarding allogeneic MSCs(M) or MSCs(AT) transplantation for the restoration of the corneal epithelium in LSCD [92,93,94,109,111,112]. Calonge et al. reported the first prospective, randomized, double-masked pilot clinical trial that tested allogeneic MSCs(M) transplantation (MSCT) in patients with severe bilateral or standard treatment-resistant LSCD, which was compared to CLET. Using the human amniotic membrane as a substrate for cell culture and cell transfer, they recorded an 82.6% overall success rate in restoring the corneal surface 12 months after surgery with no observed complications or immune rejections. The corneal epithelial phenotype improved in patients treated with MSCT in 85.7% compared to CLET at 77.8%, showing that non-epithelial SC products are also safe and effective [92,93].

Another recent clinical study conducted by Boto de Los Bueis et al. assessed autologous MSCs(AT) for ocular surface regeneration in bilateral LSCD patients. Eight patients were treated with a therapeutic procedure in which the central corneal epithelium was removed and autologous MSCs(AT) were injected into each limboconjunctival quadrant and suspended over the corneal surface. The cornea was then covered with a human amniotic membrane patch. One year after surgery, six of the eight transplantations were successful, and five patients had improved uncorrected visual acuity. Long-term follow-up (after a mean of 86.5 months) showed epithelial stability in all cases; however, improvement in all of the tested variables was only maintained in one patient. Thus, the authors concluded that the therapeutic effect varied by LSCD etiology and lessened over time [94].

### 6.2. Dry Eye Disease (DED)

DED is one of the most common chronic diseases in ophthalmology with a reported prevalence of 5–50% [113]. It is a multifactorial disease characterized by a persistently unstable and/or deficient tear film causing discomfort and/or visual impairment, which is accompanied by a variable degrees of ocular surface epitheliopathy, inflammation and neurosensory abnormalities affecting millions of people [114]. Symptoms of DED include ocular discomfort and blurred vision, which negatively impacts visual function and quality of life. Although DED is subdivided into evaporative dry eye (EDE), with excessive evaporation from the tear film, and aqueous-deficient dry eye (ADDE), with reduced tear secretion from the lacrimal gland, most often, patients have a combination of both forms of DED [115].

The current treatment methods include artificial tear replacement, local anti-inflammatory and immunosuppressive therapy, which are mainly limited to improve ocular surface discomfort and inflammation [95].

To date, there are only a few reported clinical trials in which severe DED was treated with MSC- or MSC-EV-based therapy. In an open-label, prospective, phase I clinical trial conducted by Moller-Hansen et al., seven patients affected by ADDE (due to either primary or secondary Sjögren’s syndrome) were treated with a single dose of allogeneic MSCs(AT), which was administered directly into the lacrimal gland through transconjunctival injection. The study found the treatment to be safe and effective, improving tear secretion and reducing inflammation [95].

Another study successfully used allogeneic MSCs(M) administered by intravenous injection to treat Graft-versus-host disease (GVHD)–associated DED [96]. Chronic graft-versus-host disease (cGVHD) is a serious complication of allogeneic hematopoietic SC transplantation. Ocular surface damage is one of the most common pathological manifestations in patients with cGVHD and occurs in up to 80% of patients. Clinical presentations associated with ocular cGVHD include GVHD-associated DED and cicatricial conjunctivitis, which can in advanced cases result in corneal ulcers and serious visual impairment, severely affecting the patients’ quality of life [116,117]. Effective therapy is limited to standard DED therapies, which often fails to provide a marked resolution in symptoms [118,119].

A new treatment strategy using MSCs shows promise due to their immunoregulatory effects. MSCs are known to modulate inflammatory responses by increasing and maintaining regulatory T cell activity [120,121]. An imbalance between Th1 and Th2 cytokines is suggested to drive disease progression in cGVHD [122]. Thus, MSCs might more specifically address the immunopathological mechanism behind cGVHD. MSCs were shown to induce the expansion of regulatory T cells, which may control Th1 and Th2 cytokine-mediated immunity [120,121]. In a recent study, Weng et al. explored the immunomodulatory effects of MSCs on regulatory CD8+CD28− T lymphocytes in (GVHD)-associated DED. After the intravenous injection of MSCs, symptoms improved in 12 out of 22 patients, correlating with an increased level of CD8+CD28− T cells; however, levels of CD4+CD25+ T cells did not elevate [96]. In addition, most of the patients whose symptoms improved with the MSCs treatment were able to taper and/or discontinue the immunosuppressive therapy [96].

In addition, Zhou et al. reported a successful topical use of exosomes obtained from MSCs(CB) as eye drops in a prospective clinical trial to treat 28 eyes of patients with refractory GVHD-associated DED [97]. The authors suggested that DED symptom and sign relief was a consequence of reduced inflammation due to reprogramed pro-inflammatory M1 macrophages toward the immunosuppressive M2 influencing the IL-6/IL-6R/Stat3 pathway through miRNA-204 (miR-204). This research suggests MSC-Exos as a potential treatment with miR-204 being a possible therapeutic target [97].

### 6.3. Persistent Sterile Corneal Epithelial Defect

Persistent epithelial defects (PEDs) of the cornea are caused by factors like dry eye, corneal epithelial SC deficiency, diabetes mellitus, and neurotrophic keratopathy, potentially leading to stromal degradation and corneal perforation. Treatments vary by cause and include eye patching, unpreserved artificial tears, bandage contact lenses, tarsorrhaphy, autologous serum eye drops, limbal stem cell transplantation, and amniotic membrane grafting [98].

Agorogiannis et al. presented a patient with a post-traumatic corneal PED treated with topical autologous MSCs(AT). While the initial sterile ulcer area showed little change, corneal healing began 11 days post-treatment and was completed by one month. The authors speculated that autologous MSCs(AT) might promote the healing of refractory corneal epithelial defects by either directly regenerating the corneal stroma or epithelium by MSC differentiation into corneal cells or/and by paracrine function through growth factor secretion [98].

### 6.4. Keratoconus

Cellular therapy for corneal stroma diseases such as scarring, dystrophies and ectasias is gaining clinical interest as an alternative to standard surgical procedures as corneal transplantation.

Keratoconus is a condition causing progressive corneal thinning, bulging and distortion of the cornea causing secondary vision loss due to high irregular astigmatism [123]. In advanced cases, it typically requires corneal transplantation for vision rehabilitation. Stem cell therapy aims to replace or regenerate the diseased corneal tissue directly without the need for transplantation, which in the long term has limitations such as graft survival and postoperative corneal astigmatism [124]. MSCs(AT) are promising due to their accessibility and differentiation capacity.

In 2017, Alio del Barrio and his research group conducted a phase 1 clinical study to evaluate the safety and efficacy of autologous MSCs(AT) implantation within the corneal stroma of five consecutive patients with advanced keratoconus [99]. None of these eyes had received corneal collagen cross-linking or other ophthalmic interventions in the past. No intraoperative or postoperative complications were recorded. All patients improved their visual function. Optical coherence tomography showed a slight improvement in central corneal thickness and new collagen production; however, the production was small and not homogeneously distributed along the surgical plane [99]. Therefore, in 2019, the same research group conducted another clinical study in advanced keratoconus patients using autologous MSCs(AT) with or without sheets of decellularized donor human corneal stroma [100]. Fourteen patients were selected and divided into three experimental groups. Group A patients underwent the implantation of autologous MSCs(AT) alone, group B patients received decellularized donor corneal stroma, and group C patients received decellularized donor corneal laminas with autologous MSCs(AT) at the time of surgery. No complications were observed during 1-year follow-up, showing improvements in vision and corneal thickness [100]. Confocal microscopy showed a significant increase in cell density one year after the treatment compared to preoperative density levels [101]. A long-term study from the same research group reported no complications and moderately improved uncorrected and corrected distance visual acuity 3 years after treatment [102].

## 7. Future Directions and Conclusions

The field of regenerative medicine using MSC-based therapies holds immense promise for the treatment of advanced corneal diseases causing visual impairment. This new paradigm, which focuses on tissue regeneration, may revolutionize the current clinical practice, in which treatment for corneal blindness is based on tissue replacement strategies using various corneal transplantation techniques. Thus, this new regenerative approach could overcome some of the well-known shortcomings of current standard treatments such as the scarcity of donor corneal tissues, microbiological and immunological risk factors that influence long-term graft survival. With an aging population, the need for corneal transplantation is even rising; thus, new cost-effective and more accessible long-term solutions are needed urgently.

However, several issues remain to be overcome before these new regenerative methods can be used routinely in a day-to-day clinical practice. As MSC-based therapies are classified as ATMPs, the critical quality of the final product needs to be strictly defined with the standardization and optimization of work protocols before clinical use, which need to be implemented in specialized good manufacturing practice (GMP)-accredited centers. As such, reproducible and standardized isolation, culture and differentiation protocols need to be further developed to preserve sufficient MSC content after recovery from various MSC-tissue sources. Furthermore, if MSC-EVs are considered as therapy, a better understanding of the precise therapeutic mechanisms is needed, as well as strict toxicological and microbiological studies, optimization of their therapeutic cargo, and studies that would define the pharmacological characteristics like bioavailability, targeting, pharmacokinetics, and bio-distribution. Next, another important consideration that needs to be evaluated is determining the optimal route of MSC-based therapy administration, whether it is local or systemic. In case of local delivery, the usage of scaffolds (e.g., amniotic membrane) or cell suspension strategies needs to be further defined. Moreover, in the future, corneal organoids, which are biological constructs, made out of cultured corneal epithelial tissue, stromal tissue from non-human origin, and corneal endothelial tissue shaped with 5D printing techniques to deliver customized corneas, could potentially solve donor cornea shortage and provide better therapeutic outcomes. Thus, future directions might include research that would enable more widespread availability of the treatment by providing GMP-compliant, accessible, reliable, validated, pretested, and robust MSC-based therapies for treating various advanced corneal diseases.

## Figures and Tables

**Table 1:** Human clinical studies using mesenchymal stem cell (MSC) or MSC-derived extracellular vesicle therapy for ocular surface disease and corneal regeneration.

Author [Reference]	Ophthalmic Disease	SC/EV Used	Purpose of the Study	Study Results
Calonge et al. [92,93]	LSCD	Allogeneic MSC(M)	To test whether allogeneic MSC(M) transplantation was as safe and efficient as allogeneic cultivated limbal epithelial transplantation; to improve corneal epithelial damage due to LSCD.	Improvement of central corneal epithelial phenotype. MSCT was as safe and efficacious as CLET with no adverse effects.
Boto de Los Bueis et al. [94]	LSCD	Autologous MSC(AT)	To determine the safety and feasibility of human autologous adult MSC(AT) for ocular surface regeneration in patients with bilateral LSCD.	One year after surgery, 6 of the 8 transplantations were successful, 5 patients had improved uncorrected VA. Long-term follow-up (after a mean of 86.5 months) showed epithelial stability in all cases.
Møller-Hansen et al. [95]	DED	Allogeneic MSC(AT)	To evaluate the safety and feasibility of allogeneic MSC(AT) injections into the lacrimal gland as a treatment for aqueous deficient DED.	The treatment is safe and effective, improving tear secretion and reducing inflammation.
Weng et al. [96]	cGVHD-associated DED	MSC(M)	To investigate the efficacy of intravenous application of MSC(M) for the treatment of cGVHD-associated DED and assess the immunomodulatory effects of MSC(M) on regulatory CD8(+)CD28(−) T lymphocytes.	Symptoms improved in half of the treated patients. In those who responded to treatment, there was an increase in CD8(+)CD28(−) T lymphocyte levels, while no effect on CD4(+)CD25(+) was observed. Patients who were effectively treated had higher levels of Th1 cytokines and lower levels of Th2 cytokines.
Zhout et al. [97]	cGVHD-associated DED	MSC(UC)-Exo	To test whether eye drops containing MSC(UC)-Exo could be effective in treating symptoms and signs of cGVHD-associated DED.	Relief and improvement of symptoms and signs. Reduced inflammation due to reprogramed pro-inflammatory M1 macrophages toward the immunosuppressive M2 influencing the IL-6/IL-6R/Stat3 pathway through miR-204.
Agorogiannis et al. [98]	PED	Autologous MSC(AT)	To determine whether topical application of autologous MSC(AT) will affect the healing of post-traumatic PED.	Topical application of autologous MSC(AT) promoted healing of epithelial defects.
Alio del Barrio et al. [99]	Keratoconus	Autologous MSC(AT)	To evaluate the safety and efficacy of autologous MSC(AT) implantation within the corneal stroma of patients with advanced keratoconus.	Treatment improved VA and CCT. Production of new collagen was small. No complications were recorded.
Alio et al. [100]; El Zarif et al. [101,102]	Keratoconus	Autologous MSC(AT)	To evaluate safety and efficacy of autologous MSC(AT) implantation with or without sheets of decellularized donor human corneal stroma in treating patients with advanced keratoconus.	At the 1-year follow-up, no complications were noted with improvements in VA, CCT, and increased cell density. Three years post-treatment, there were still no complications, and moderate improvements in uncorrected and corrected distance VA persisted.

Abbreviations: SCs—stem cells; LSCD—limbal stem cell deficiency; MSCs(M)—bone marrow-derived mesenchymal stem cells; MSCT—mesenchymal stem cell transplantation; CLET—cultivated limbal epithelial transplantation; MSCs(AT)—adipose tissue derives mesenchymal stem cells; VA—visual acuity; DED—dry eye disease; PED—persistent epithelial defect; cGHVD—chronic guest versus host disease; MSC(UC)—exo-exosomes obtained from human umbilical cord-derived mesenchymal stem cells; IL—interleukin; CCT—central corneal thickness.

## Data Availability

No new data were created.
